# The Phosphatase PHLPP2 Plays a Key Role in the Regulation of Pancreatic Beta-Cell Survival

**DOI:** 10.1155/2020/1027386

**Published:** 2020-01-03

**Authors:** Marta Letizia Hribal, Elettra Mancuso, Gaetano Paride Arcidiacono, Annalisa Greco, Donatella Musca, Teresa Procopio, Mariafrancesca Ruffo, Giorgio Sesti

**Affiliations:** ^1^Department of Medical and Surgical Sciences, University Magna Græcia of Catanzaro, Catanzaro, Italy; ^2^Department of Medicine, University of Padua, Padua, Italy; ^3^Department of Medicine, Ausl of Bologna, Bologna, Italy; ^4^Department of Clinical and Molecular Medicine, University of Rome La Sapienza, Rome, Italy

## Abstract

Currently available antidiabetic treatments fail to halt, and may even exacerbate, pancreatic *β*-cell exhaustion, a key feature of type 2 diabetes pathogenesis; thus, strategies to prevent, or reverse, *β*-cell failure should be actively sought. The serine threonine kinase Akt has a key role in the regulation of *β*-cell homeostasis; among Akt modulators, a central role is played by pleckstrin homology domain leucine-rich repeat protein phosphatase (PHLPP) family. Here, taking advantage of an in vitro model of chronic exposure to high glucose, we demonstrated that PHLPPs, particularly the second family member called PHLPP2, are implicated in the ability of pancreatic *β* cells to deal with glucose toxicity. We observed that INS-1 rat pancreatic *β* cell line maintained for 12–15 passages at high (30 mM) glucose concentrations (INS-1 HG) showed increased expression of PHLPP2 and PHLPP1 both at mRNA and protein level as compared to INS-1 maintained for the same number of passages in the presence of normal glucose levels (INS-1 NG). These changes were paralleled by decreased phosphorylation of Akt and by increased expression of apoptotic and autophagic markers. To investigate if PHLPPs had a casual role in the alteration of INS-1 homeostasis observed upon chronic exposure to high glucose concentrations, we took advantage of shRNA technology to specifically knock-down PHLPPs. We obtained proof-of-concept evidence that modulating PHLPPs expression may help to restore a healthy *β* cell mass, as the reduced expression of PHLPP2/1 was accompanied by a recovered balance between pro- and antiapoptotic factor levels. In conclusion, our data provide initial support for future studies aimed to identify pharmacological PHLPPs modulator to treat beta-cell survival impairment. They also contribute to shed some light on *β*-cell dysfunction, a complex and unsatisfactorily characterized phenomenon that has a central causative role in the pathogenesis of type 2 diabetes.

## 1. Introduction

Type 2 diabetes (T2D) is a complex disease, brought about by the combination of abnormalities in both the production and the function of the pancreatic hormone insulin [1]. Although classically these two defects were seen as separate entities, in the last decades, it has become evident that they share common pathogenetic mechanisms, with insulin regulating not only glucose utilization from peripheral target tissues, but also its own synthesis and secretion as well as the maintenance of an adequate *β*-cell mass [1–3]. Notably, while impaired insulin action in peripheral tissue—the so-called “insulin resistance”—remains fairly constant as the disease progresses, *β*-cell function worsens continuously with time in diabetic patients, as a consequence of the persisting exposure to damaging factors, such as high glucose concentrations (glucose toxicity), increased levels of circulating free fatty acids (lipotoxicity), and proinflammatory cytokines (chronic inflammation) [2–5]. Furthermore, currently available antidiabetic treatments fail to halt, and may even exacerbate, pancreatic *β*-cell exhaustion; thus, despite promising observations with molecules belonging to the more recently introduced therapeutic classes [6], strategies to prevent, or reverse, *β*-cell failure should still be actively sought. There are two primary components to *β*-cell dysfunction in T2D: impaired insulin secretion and reduced *β*-cell mass. In adult humans, the rate of new *β*-cell formation is low, and the maintenance of an adequate mass is achieved mainly throughout a tight regulation of apoptotic rates [7]. The serine threonine kinase Akt, also known as protein kinase B (PKB), has a key role in the regulation of *β*-cell homeostasis. Akt exists in three isoforms that are considered indistinguishable in their domain architecture and upstream regulation but are nonredundant in their expression patterns and biological functions [8, 9]. Specifically, all three isoforms have been detected in pancreatic *β*-cells; with studies in knock-out mouse models suggesting that Akt1 regulates mainly *β*-cell survival, Akt2 is required to modulate the insulin secretory response, while Akt3 loss does not appear to significantly alter either *β*-cell mass or function [9]. The three Akt isoforms are activated by sequential phosphorylation at two key sites; the phosphorylation of the first residue, located in a segment called the activation loop (Threonine 308/309/307 in Akt1/2/3, respectively), triggers the phosphorylation of a site located in the carboxyl-terminal domain, termed the hydrophobic phosphorylation motif (Serine 473/474/472 in Akt1/2/3 respectively) [8]. We and others have reported decreased Akt activation upon exposure to glucotoxicity, lipotoxicity, and/or chronic inflammation [4, 9–14]. Akt inhibition is mediated by dephosphorylation by two protein phosphatases: protein phosphatase 2A that acts on the threonine residue [15] and pleckstrin homology domain leucine-rich repeat protein phosphatase (PHLPP) family targeting the serine residue [16–18]. PHLPP proteins appear to be ubiquitously expressed, with particularly high levels in the brain, and their increased expression has been reported in numerous cancer cell lines, including cancers of the brain, breast, lung, prostate, and ovary. The PHLPP family comprises two members: PHLPP1, which in turn exists in two splice variants, (*α* and *β*), and PHLPP2 (also known as PHLPPL). The three isozymes share very similar domain structure but have a certain degree of substrate specificity, with PHLPP1 preferentially targeting Akt2 and 3 and PHLPP2 showing a higher affinity toward Akt1 and 3 [18]. A few years ago, we have shown increased PHLPP1 expression in adipose tissue and skeletal muscle biopsies from obese, insulin-resistant subjects and hypothesized that PHLPPs may represent an additional player in insulin resistance [14]. Here, we investigated if PHLPPs may be implicated in the ability of pancreatic *β* cells to deal with chronic exposure to high glucose concentrations in an *in vitro* model. We also aimed to obtain proof-of-concept evidence that modulating PHLPPs expression may help to restore a healthy *β* cell mass.

## 2. Methods

### 2.1. Cell Culture and Adenoviral Infection

Rat pancreatic *β*-cells lines were maintained at 37°C with 5% CO_2_ for 12–15 passages in RPMI 1640 cell medium (Sigma-Aldrich, Milan, Italy), supplemented with 50 *μ*M *β*-mercaptoethanol, 10% (vol/vol) fetal bovine serum, and 1% (vol/vol) penicillin/streptomycin [19], and containing 11.1 mM (INS-1 NG) or 30 mM (INS-1 HG) glucose. Adenoviral infection was carried out, as previously described [20] incubating 50–60% confluent INS-1 HG with increasing quantities of a 1 : 1 mixture containing Ad-U6-rat-PHLPP1-shRNA and Ad-U6-rat-PHLPP2-shRNA (viral titer 3.7 × 10^10^ PFU/ml, Vector Biosystems, Malvern, PA, USA) or Ad-U6 scrambled-shRNA (mock-infected control cells) for 7 hrs and 30 minutes. Cells were then washed to remove virus and serum-starved or maintained in fresh complete growth medium for 48 hrs, depending on the specific experimental requirements.

### 2.2. Total RNA Extraction and Real-Time Reverse Transcription Quantitative Polymerase Chain Reaction (RT-qPCR)

Total RNA was extracted from INS-1 NG and INS-1 HG using Trizol (Life Technologies, Gaithersburg, MD), reverse transcribed and analyzed by RT-qPCR using a Power SYBR Green PCR Master Mix (Applied Biosystems, Foster City, CA). Results were normalized to *β*-actin levels according to the Livak method, as previously described [21–23]. Primers sequences are available upon request.

### 2.3. Insulin Stimulation and Western Blot Analysis

To assess insulin-stimulated protein phosphorylation, INS-1 were serum-starved for 48 hrs with FBS-free medium containing bovine serum albumin and glucose at the appropriate concentrations; human insulin (10^−7^ M) was then added, when indicated, for 7 minutes before cell lysis in a buffer containing 1.5% NP-40. Cell lysates were processed and analyzed by Western Blot, according to previously established methods [14, 20]. A home-made primary antibody generated and validated by our research group [14] was employed to detect PHLPP1 levels; anti-PHLPP2 antibody was obtained from Abcam (Cambridge, United Kingdom). The following primary antibodies were purchased from Cell Signaling Technology (Danvers, MA, USA): anti-Bad, anti-Bcl-xL, anti-cleaved caspase-3, anti-LC3-II, anti-total and phospho Akt, anti-total and phospho FoxO1, and anti-total and phospho mTor. Equal protein loading was confirmed by reblotting the membranes with monoclonal antibody against *β*-actin (Sigma-Aldrich; Milan, Italy); p-Akt/Akt, p-FoxO1/FoxO1, and pmTor/mTor ratios were calculated to analyze the relative phosphorylation levels. Densitometric analysis was performed using a ImageJ software (NIH, USA).

### 2.4. Insulin Secretion Assay

INS-1 cells were seeded in 24-multiwell plates at a density of 10^5^ cells/well in growth medium containing 11.1 mM or 30 mM glucose, as appropriate. Twenty hours before the insulin secretion assay, cells were switched to a medium containing 5 mM glucose; the medium was then replaced with a glucose-free Krebs phosphate buffer for 2 hrs. INS-1 was then incubated in the presence of increasing concentrations of glucose in fresh Krebs buffer for 20 minutes. Cell media were removed and diluted to assess insulin concentration with a specific rat insulin Elisa assay (Mercodia, Uppsala, Sweden).

### 2.5. Statistical Analysis

All results were calculated as mean fold variation (±SD) over the appropriate control point. Statistical differences were assessed by Student's *t* test or ANOVA as indicated. A *p* value ≤0.05 was considered statistically significant. Analyses were performed with GraphPad Prism version 8.2.0 software (San Diego, CA, USA).

## 3. Results

### 3.1. Chronic Exposure to High Glucose Concentrations Results in a Significant Increase of PHLPP2 and PHLPP1 Expression

**Figure 1:**
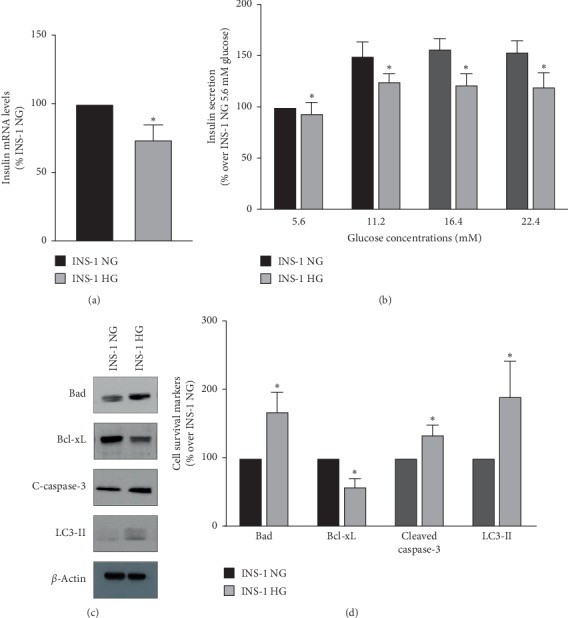
INS-1 cells maintained for 12–15 passages at 30 mM glucose concentrations show reduced insulin synthesis and secretion and altered expression of prosurvival markers. INS-1 was maintained at high (30 mM, INS-1 HG) or normal (11.2 mM, INS-1 NG) glucose concentrations for 12–15 passages. (a) Insulin mRNA levels were assessed by real-time RT-PCR; *n* = 6, ^*∗*^indicates significant (*p* < 0.05) differences for INS-1 NG vs INS-1 HG. (b) Glucose-stimulated insulin secretion was assessed by measuring with a specific Elisa kit insulin concentration in medium obtained from INS-1 NG and INS-1 HG exposed to increasing glucose concentrations (5.6, 11.2, 15.6, and 22.4 mM) for 20 minutes; *n* = 4, ^*∗*^indicates significant (*p* < 0.05) differences for INS-1 NG vs INS-1 HG. (c) Representative western blot images of cell survival markers levels in INS-1 HG and INS-1 NG: Bad (upper panel), Bcl-xL (middle-upper panel), cleaved caspase-3 (middle-lower panel), and LC3-II (lower panel). (d) Graph of the mean changes of densitometric values of cell survival markers in INS-1 NG and INS-1 HG; *n* = 5–10, ^*∗*^indicates significant (*p* < 0.05) differences for INS-1 NG vs INS-1 HG.

To mimic chronic exposure to high glucose levels, we cultured INS-1 rat pancreatic *β*-cells at 30 mM glucose for 12–15 passages (INS-1 HG). This glucose concentration has been previously shown to efficaciously induce glucose toxicity in pancreatic *β*-cell lines that require culture media containing 11.2 mM glucose for their normal growth [24]. As compared to INS-1 cells maintained for the same number of passages at normal glucose concentrations (INS-1 NG), the functionality of INS-1 HG was significantly impaired, as demonstrated by the decreased insulin mRNA levels (Figure 1(a), *n* = 6, *p*=0.0001, INS-1 HG vs INS-1 NG) and the hampered glucose-stimulated insulin secretion (Figure 1(b), *n* = 4, *p*=0.0001, by 2-way ANOVA for the secretion curve of INS-1 HG vs INS-1 HG). Furthermore, the balance between pro- and antiapoptotic Bcl family protein expression appeared altered in INS-1 HG (Figures 1(c) and 1(d), *n* = 4, *p*=0.04, INS-1 HG vs INS-1 NG). The activation of apoptotic pathways in INS-1 HG was suggested also by the increased levels of cleaved caspase-3 (Figures 1(c) and 1(d), *n* = 10, *p*=0.0001, for INS-1 HG vs INS-1 NG). In addition, we observed a two-fold increase in the amount of LC3-II protein, which hinted to a higher activation of autophagic pathways in INS-1 HG cells as compared to INS-1 NG (Figures 1(c) and 1(d), *n* = 6, *p*=0.0001, for INS-1 HG vs INS-1 NG).

**Figure 2:**
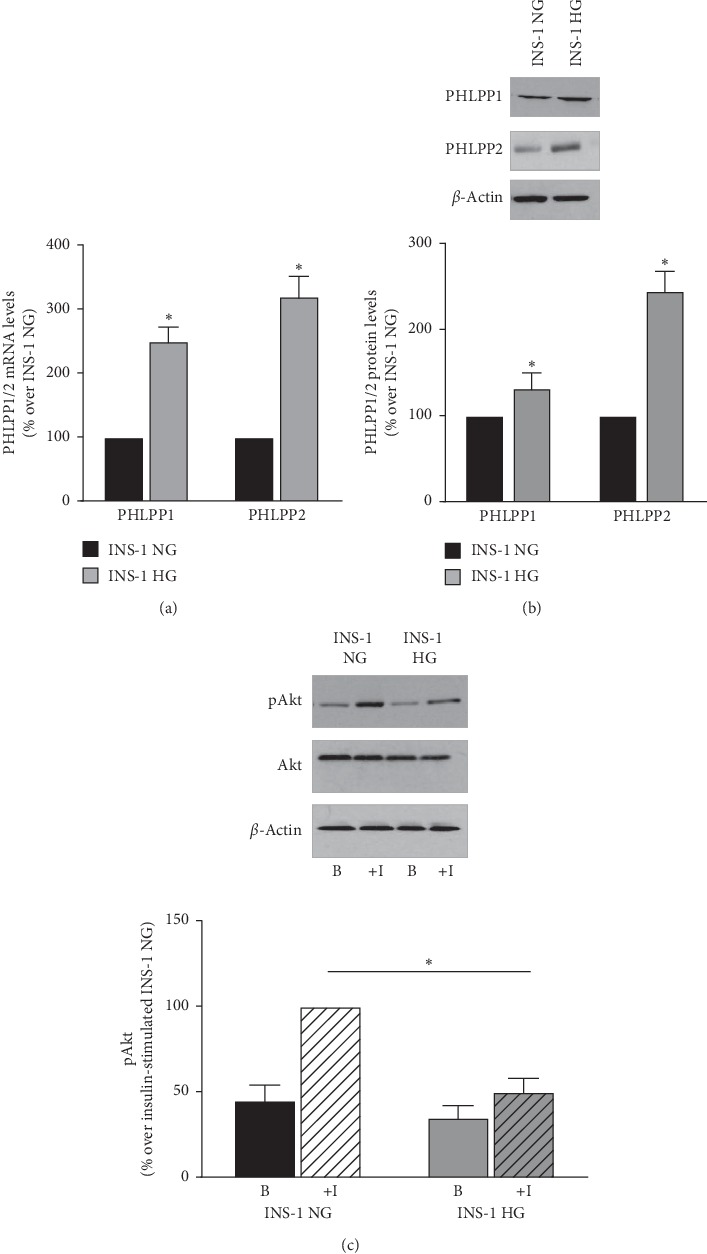
PHLPP1 and PHLPP2 expression is increased and Akt phosphorylation is reduced in INS-1 HG. (a) PHLPP1 and PHLPP2 mRNA levels were assessed by real-time RT-PCR (A), *n* = 5, ^*∗*^indicates significant (*p* < 0.05) differences for INS-1 NG vs INS-1 HG; (b) representative Western Blot images of PHLPP1 (upper panel) and PHLPP2 (lower panel) levels in INS-1 NG and INS-1 HG. Graphs of the mean changes over INS-1 NG values of the densitometric values of PHLPP1/2 expression obtained in 4 independent experiments and normalized for *β*-actin levels; ^*∗*^indicates significant (*p* < 0.05) differences for INS-1 NG vs INS-1 HG. (c) Representative Western Blot images of Akt phosphorylation on the Serine 473 residue (upper panel) and of total Akt levels (middle panel) in INS-1 NG and INS-1 HG stimulated (+I) or not (b) with 10^−7^ M insulin. Graphs of the mean changes over insulin-stimulated INS-1 NG values of the densitometric values of pAkt obtained in 4 independent experiments and normalized for total Akt levels. ^*∗*^indicates significant (*p* < 0.05) differences for insulin-stimulated INS-1 HG vs insulin-stimulated INS-1 NG.

These functional changes were paralleled by a significant increase in PHLPP2 expression at both mRNA (Figure 2(a), +320 ± 35%, *p*=0.0001, for INS-1 HG vs INS-1 NG *n* = 5) and protein level (Figure 2(b) +245 ± 25%, *p*=0.0001, for INS-1 HG vs INS-1 NG, *n* = 4) in INS-1 HG as compared to INS-1 NG. Similar, but less evident, changes were observed for PHLPP1 expression, which showed a 250 ± 22% increase in mRNA levels (Figure 2(a), *p*=0.0001 for INS-1 HG vs INS-1 NG, *n* = 5) and 132 ± 20% increase in protein levels (Figure 2(b), *p*=0.047 for INS-1 HG vs INS-1 NG, *n* = 4).

Since PHLPP dephosphorylates and inactivates Akt kinase [16–18], we assessed Akt phosphorylation on the activation loop residue, Ser473, which is directly targeted by PHLPPs. As shown in Figure 2(c), basal and insulin-stimulated Akt phosphorylation on Ser473 was reduced in INS-1 HG as compared to INS-1 NG (upper panel, *p* = 0.049 for basal INS-1 HG vs basal INS-1 NG; *p*=0.0001 for insulin-stimulated INS-1 HG vs insulin-stimulated INS-1 NG, *n* = 6). Interestingly, we observed a slight, not significant, decrease of total Akt expression in INS-1 HG (Figure 2(c), middle panel). The reduction of insulin-stimulated Akt phosphorylation was statistically significant when normalized taking into account the reduced total Akt levels (Figure 2(c), *p*=0.0001, for insulin-stimulated INS-1 HG vs insulin-stimulated INS-1 NG, *n* = 6).

### 3.2. Infection with shRNA Constructs Against PHLPP2/1 Resulted in a Significant Knock-Down of the Expression of Both Isoenzymes

**Figure 3:**
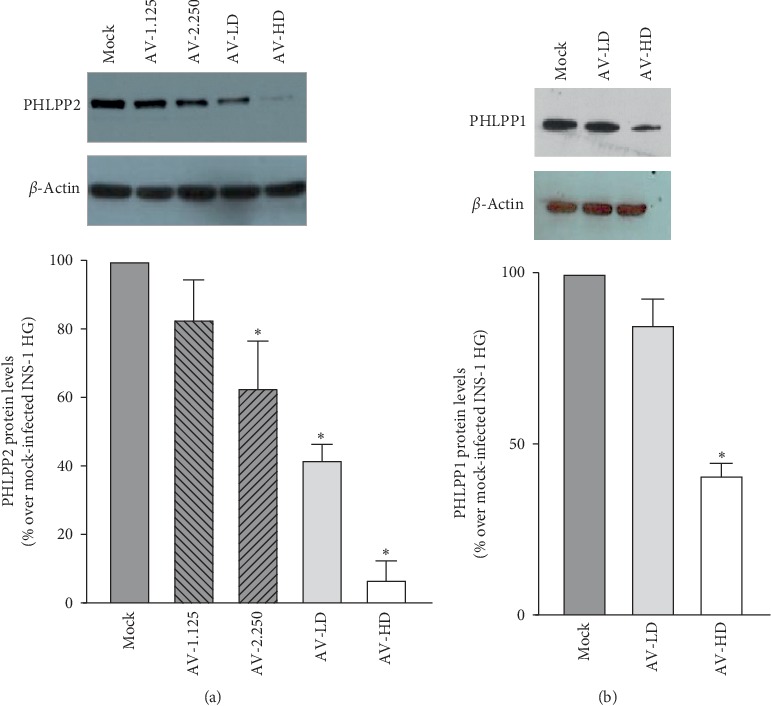
Infection with shRNA constructs against PHLPP2/1 results in a significant knock-down of the expression of both isoenzymes. (a) Representative Western Blot images of PHLPP2 levels in INS-1 HG infected with increasing concentrations of the adenoviral vectors encoding for shRNA against PHLPP1/2 : 1.125 × 10^5^ PFU (AV1.125); 2.250 × 10^5^ PFU (AV2.250); 4.5 × 10^5^ PFU (AV-LD); and 9 × 10^5^ PFU (AV-HD). Control mock-infected cells were infected with empty AV constructs. Graphs of the mean changes over mock-infected INS-1 HG values of the densitometric values of PHLPP2 expression obtained in 4 independent experiments and normalized for *β*-actin levels; ^*∗*^indicates significant (*p* < 0.05) differences for AV-infected INS-1 HG vs mock-infected INS-1 HG. (b) Representative Western Blot images of PHLPP1 levels in INS-1 HG infected with 4.5 × 10^5^ PFU (AV-LD) or 9 × 10^5^ PFU (AV-HD) of the of the adenoviral vectors encoding for shRNA against PHLPP1/2 as compared to control mock-infected INS-1 HG cells. Graphs of the mean changes over mock-infected INS-1 HG values of the densitometric values of PHLPP1 expression obtained in 4 independent experiments and normalized for *β*-actin levels; ^*∗*^indicates significant (*p* < 0.05) differences for AV-infected INS-1 HG vs mock-infected INS-1 HG.

To investigate if PHLPPs had a casual role in the alteration of INS-1 homeostasis observed upon chronic exposure to high glucose concentrations, we took advantage of shRNA technology to specifically knock-down PHLPPs, employing a previously validated protocol [20]. We choose to concurrently downregulate both PHLPP family member, since the expression of both appeared increased upon chronic exposure to high glucose concentrations, and specifically reducing only one isoenzyme may have induced compensatory overexpression of the cognate protein, confounding the data interpretation. To this end, we employed a mixture containing two adenoviral vectors encoding for a shRNA against PHLPP1 or against PHLPP2 in a 1 : 1 ratio. We initially performed a dose-response curve and observed that a dose of at least 4.5 × 10^5^ PFU was required to consistently reduce PHLPP2 levels; doubling this dose, we obtained an almost complete PHLPP2 knock-down (Figure 3(a), *p*=0.0001, as compared to mock-infected INS-1 HG cells). We then tested if these two concentrations resulted in PHLPP1 expression inhibition and observed a 15% reduction of PHLPP1 levels with the 4.5 × 10^5^ PFU (*p*=0.04) and a 85% decrease with the 9 × 10^5^ PFU dose (*p*=0.0001) (Figure 3(b)). Higher viral doses resulted in a notable and rapid decrease in cell viability; all subsequent experiments were thus carried out with the above-mentioned quantities that will be referred to as low dose (AV-LD) and high dose (AV-HD).

### 3.3. shRNA-Mediated PHLPP2 and PHLPP1 Knock-Down Restores Insulin Signaling in INS-1 HG

**Figure 4:**
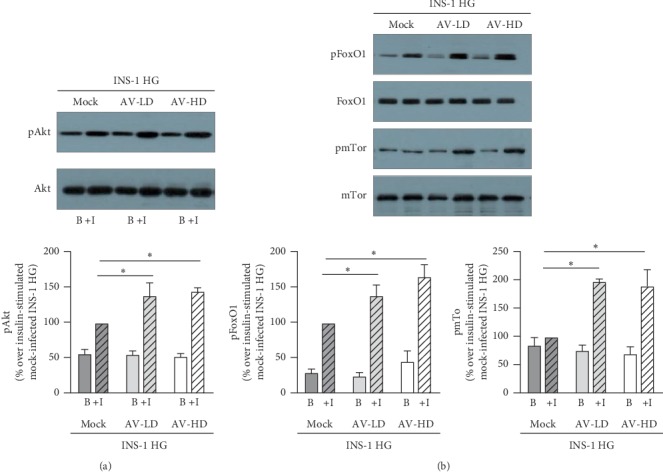
PHLPP2/1 knock-down results in a significant improvement of insulin-stimulated phosphorylation of Akt, FoxO1, and mTor. (a) Representative western blot images of Akt phosphorylation on the Serine 473 residue (upper panel) and of total Akt levels (middle panel) in mock-infected INS-1 HG, INS-1 HG AV-LD, INS-1 HG AV-HD-stimulated (+I), or not (b) with 10^−7^ M insulin. Graphs of the mean changes over insulin-stimulated mock-infected INS-1 HG values INS-1 NG values of the densitometric values of pAkt obtained in 3 independent experiments and normalized for total Akt levels; ^*∗*^indicates significant (*p* < 0.05) differences for AV-infected INS-1 HG vs mock-infected INS-1 HG. (b) Representative Western Blot images of FoxO1 phosphorylation (upper panel), total FoxO1 levels (upper-middle panel), mTor phosphorylation (lower-middle panel), total mTor levels (lower panel) in mock-infected INS-1 HG, INS-1 HG AV-LD, INS-1 HG AV-HD-stimulated (+I), or not (b) with 10^−7^ M insulin. Graphs of the mean changes over insulin-stimulated mock-infected INS-1 HG values and INS-1 NG values of the densitometric values of pFoxO1 or pmTor obtained in 3–5 independent experiments and normalized for total levels of the unphosphorylated protein; ^*∗*^indicates significant (*p* < 0.05) differences for AV-infected INS-1 HG vs mock-infected INS-1 HG.

Next, we evaluated if in INS-1 HG infected with either AV-LD or AV-HD, the reduction of PHLPPs expression resulted in a restored activation of Akt signaling pathway upon insulin stimulation. We observed that insulin-stimulated Akt phosphorylation levels were 1.39 and 1.45 fold higher in INS-1 HG AV-LD and INS-1 HG AV-HD, respectively, as compared to mock-infected control INS-1 HG (*p*=0.0022, for INS-1 HG AV-LD vs mock-infected INS-1 HG and *p*=0.0003 for INS-1 HG AV-HD vs mock-infected INS-1 HG, *n* = 4, Figure 4(a)). The increased Akt phosphorylation levels were mirrored by significantly increased insulin-stimulated phosphorylation of two Akt substrates that have been suggested to play key roles in the regulation of specific *β*-cell function: FoxO1 transcription factor (*p*=0.0018 for INS-1 HG AV-LD vs mock-infected INS-1 HG and *p*=0.0001 for INS-1 HG AV-HD vs mock-infected INS-1 HG, *n* = 3, Figure 4(b)), and mTor kinase (*p*=0.0008 for INS-1 HG AV-LD vs mock-infected INS-1 HG and *p*=0.0001 for INS-1 HG AV-HD vs mock-infected INS-1 HG, *n* = 35 (Figure 4(b)).

shRNA-mediated PHLPP2 and PHLPP1 knock-down reestablishes the balance of apoptotic factor and decreases the activation of autophagic pathways.

**Figure 5:**
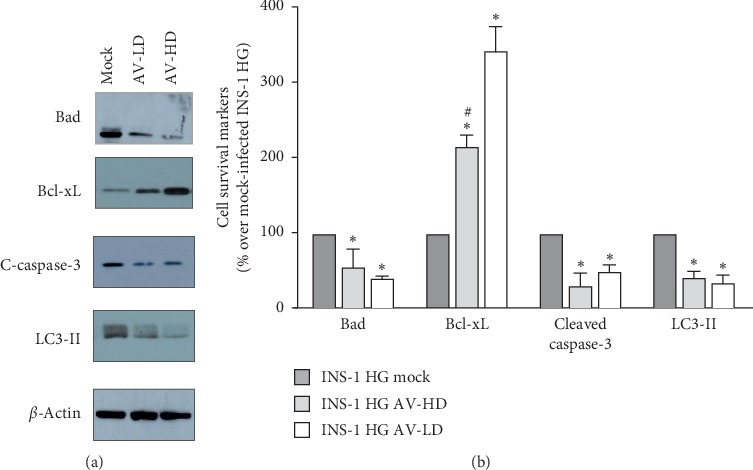
PHLPP2/1 knock-down results in a significant improvement of cell survival markers expression profile. (a) Representative western blot images of cell survival markers levels in mock-infected INS-1 HG, INS-1 HG AV-LD, and INS-1 HG AV-HD: bad (upper panel), Bcl-xL (middle-upper panel), cleaved caspase-3 (middle-lower panel), and LC3-II (lower panel). (b) Graph of the mean changes of densitometric values of cell survival markers levels in mock-infected INS-1 HG, INS-1 HG AV-LD, and INS-1 HG AV-HD; *n* = 3–5; ^*∗*^indicates significant (*p* < 0.05) differences for AV-infected INS-1 HG vs mock-infected INS-1 HG, ^#^indicates significant (*p* < 0.05) differences for INS-1 HG AV-LD vs INS-1 HG AV-HD.

We thus assessed if the reduced PHLPPs levels and the restored activation of Akt signaling pathway resulted in improved cell viability. To this end, we measured Bad and Bcl-xL expression and observed that the pro/antiapoptotic factor balance was repristinated in both INS-1 AV-LD and INS-1 HG AV-HD as compared to mock-infected INS-1 HG Bad: (*p*=0.0007 for INS-1 HG AV-LD vs mock-infected INS-1 HG and *p*=0.0001 for INS-1 HG AV-HD vs mock-infected INS-1 HG, *n* = 4, Bcl-xL *p*=0.0008 for both INS-1 HG AV-LD and INS-1 HG AV-HD vs mock-infected INS-1 HG, *n* = 3, Figure 5(a)). In keeping with these results, we observed also a significantly decreased caspase-3 cleavage in INS-1 HG infected with either AV-LD or AV-HD (*p*=0.0001 for INS-1 HG AV-LD vs mock-infected INS-1 HG and *p*=0.0002 for INS-1 HG AV-HD vs mock-infected INS-1 HG, *n* = 4). Notably, while the effects obtained with the higher AV dose were of a higher magnitude as far as Bcl proteins expression was concerned, INS-1 HG AV-LD showed a lower caspase activity than cells infected with the higher AV dose; this may reflect a PHLPP-independent effect and be related to the AV itself (Figure 5(b)). The restored prosurvival profile was paralleled by decreased expression of the LC3-II (*p*=0.0001 for both INS-1 HG AV-LD and INS-1 HG AV-HD vs mock-infected INS-1 HG, *n* = 5, Figures 5(a) and 5(b)).

shRNA-mediated PHLPP2 and PHLPP1 knock-down was not sufficient to restore insulin mRNA levels or glucose-stimulated insulin secretion.

**Figure 6:**
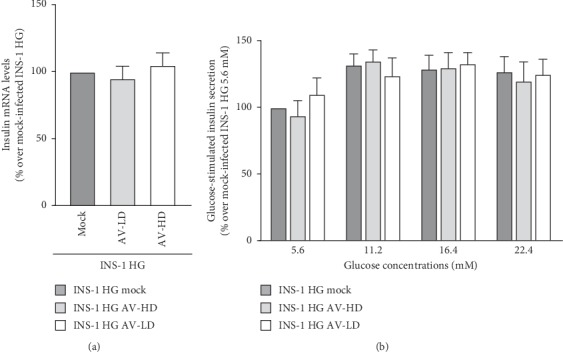
PHLPP2/1 knock-down does not restore insulin synthesis and secretion in INS-1 HG. (a) Insulin mRNA levels were assessed by real-time RT-PCR in mock-infected INS-1 HG, INS-1 HG AV-LD, and INS-1 HG AV-HD: *n* = 4. (b) Glucose-stimulated insulin secretion was assessed by measuring with a specific Elisa kit insulin concentration in medium obtained mock-infected INS-1 HG, INS-1 HG AV-LD, and INS-1 HG AV-HD exposed to increasing glucose concentrations (5.6, 11.2, 15.6, and 22.4 mM) for 20 minutes; *n* = 4.

We assessed if PHLPPs knock-down also resulted in a recovered INS-1 HG functionality. We thus measured INS-1 mRNA expression levels by real-time RT-PCR and observed no significant increase in cells infected with neither AV dose as compared to mock-infected cells (Figure 6(a), *n* = 3). Similarly, glucose-stimulated insulin secretion remained impaired in knocked down cells as the amount of insulin released in response to increasing glucose concentration was no different between INS-1 HG AV-LD, INS-1 HG AV-HD, and mock-infected INS-1 HG (Figure 6(b), *n* = 4).

## 4. Conclusions

In the present study, we report that the altered pancreatic *β*-cell homeostasis observed upon chronic exposure to 30 mM glucose is paralleled by increased expression of PHLPPs, with a consequent reduction of the phosphorylation levels of their primary target, the serine threonine kinase Akt. Interestingly, knocking-down PHLPPs, throughout adenoviral-mediated shRNA delivery, we were able to restore a prosurvival profile in INS-1 HG cells chronically exposed to high glucose levels. Specifically, the ratio between the proapoptotic factor Bad and its prosurvival counterpart Bcl-xL went back to the value measured in healthy INS-1 NG cells. Bad levels have been suggested to be directly regulated by Akt pathway [25], and indeed, we observed significantly increased phosphorylation of Akt and of its major antiapoptotic effector FoxO1 [26] in INS-1 HG infected with the adenoviral constructs encoding for specific shRNA sequences against PHLPP2 and PHLPP1. The insulin-stimulated activation of another Akt substrate mTor was also significantly improved when PHLPPs expression was knocked down. mTor senses nutrient availability and regulates cell homeostasis, and it has been suggested that its loss impairs *β*-cells homeostasis as well as insulin sensitivity in peripheral tissues [27]. Among the intracellular processes controlled by mTor, autophagy as gained attention as a possible player in the survival of pancreatic *β*-cells with conflicting data showing, in different experimental models, pro- or antiapoptotic effects of an increased activation of autophagic pathways [28–30]. In our model, the recovered prosurvival profile was paralleled by decreased expression of the autophagic marker LC3-II, supporting the hypothesis of a negative impact of a disproportionated activation of autophagic pathways on cell survival.

At odds with the restored cell survival profile, efficient glucose-stimulated insulin secretion and synthesis were not recovered by INS-1 HG infected with adenoviral vectors carrying the shRNA sequences against PHLPP2/1. It has been reported that Akt isoforms have different roles in pancreatic *β*-cells, with Akt1 mainly controlling cell survival and Akt2 mostly involved in the regulation of insulin secretion [9], and it has also been suggested that PHLPP family members possess a selective preferences toward Akt isoforms, as PHLPP2 seems to prefer Akt1 and PHLPP1 favors Akt2, even if this may depend on the predominantly expressed substrate isoform [17, 18]. Since we obtained an almost complete silencing of PHLPP2 in INS-1 cells infected with the highest adenoviral concentration (INS-1 HG AV-HD), while the maximal reduction of PHLPP1 achieved was around 65%, it may be possible to hypothesize that Akt2 function, and consequently insulin secretion and synthesis, was less efficiently restored. However, the observation that no significant difference was observed when comparing INS-1 HG AV-HD, expressing 45% of PHLPP1 with respect to mock-infected INS-1 HG, with cells infected with a lower adenoviral titer with PHLPP1 levels around 85% of those observed in mock INS-1 HG (INS-1 HG AV-LD), renders this explanation quite unlikely and rather points to the possibility that glucose toxicity may more profoundly damage *β*-cell function than *β*-cell survival, causing a depletion of insulin deposits that may not be restored with a short-term improvement of Akt activation [31, 32]. Indeed, we believe that any attempt to restore *β*-cell homeostasis in a *in vivo* setting should not overlook the important difference among the mechanisms regulating *β*-cell mass and those regulating specific *β*-cell functions such as insulin synthesis and secretion. The lack of significant differences between INS-1 HG AV-LD and INS-1 HG AV-HD cells for the majority of functional and molecular read-out analyzed also suggests that PHLPP2 may play a more pivotal role than the cognate protein, PHLPP1, in the dysregulation of beta-cell survival. Our data thus contribute a small piece of knowledge to the comprehension of the specific functions of PHLPP family members and to the less explored mechanisms regulating their own expressions [18, 33]. Interestingly, a few years ago, we and others showed a specific increase of PHLPP1, with unaltered PHLPP2 levels, in adipose tissue and skeletal muscle biopsies of obese, insulin-resistant individuals [14, 34]; these data have been more recently confirmed by Behera et al. in a high-fat fed animal model [35]. Here, we report a significant increase of both isoenzymes upon exposure to high glucose concentrations; however, PHLPP2 showed the larger and more statistical sound changes. The results of the shRNA-mediated inhibition experiments confirm a preeminent role of PHLPP2 in the regulation of pancreatic *β*-cells homeostasis.

Furthermore, in the older study, we did not observe any direct correlation between PHLPP1 expression and glucose levels, while the agent of the increased expression appeared to be insulin levels. In contrast in the present study, PHLPPs levels were increased in response to high glucose concentration, even if our data do not allow to establish if glucose is able to directly promote PHLPP transcription or transduction or if the increase in PHLPP protein levels was mediated by indirect mechanisms [14]. Nonetheless our results underlie that PHLPPs possess a specificity that has not been fully explored to date and may be differentially regulated in different tissue. Clarifying this point is mandatory in order to exploit these phosphatases as possible pharmacological targets.

In conclusion, our data provide initial support for future studies aimed to identify pharmacological PHLPPs modulator to treat beta-cell survival impairment. They also contribute to shed some light on *β*-cell dysfunction, a complex and unsatisfactorily characterized phenomenon that has a central causative role in the pathogenesis of type 2 diabetes.

## Data Availability

All data used to support the findings of the study are included within the manuscript.
